# Cancer Stem Cells' Biomarker ALDH1&2 Increased Expression in Erosive Oral Lichen Planus Compared to Oral Leukoplakia

**DOI:** 10.7759/cureus.44278

**Published:** 2023-08-28

**Authors:** Vasileios Zisis, Nikolaos N Giannakopoulos, Marc Schmitter, Athanasios Poulopoulos, Dimitrios Andreadis

**Affiliations:** 1 Prosthodontics, Julius-Maximilians-Universität, Würzburg, DEU; 2 Oral Medicine/Pathology, Aristotle University of Thessaloniki, Thessaloniki, GRC

**Keywords:** oral leukoplakia, skin inflammation, aldh, oral lichenoid reaction, oral lichen planus

## Abstract

Introduction: ALDH1&2 has been considered an oral cancer stem cell (CSC) marker. Oral carcinogenesis is a process that usually passes through oral potentially malignant disorders (OPMD). Oral lichen planus (OLP) consists of immune-related chronic disorders that have been included in the OPMDs due to their possible transformation into oral cancer. The aim of this study was to investigate the early presence of ALDH1&2 in OLP compared to early oral leukoplakias (OL), especially mildly and non-dysplastic OL.

Materials and methods: The study type is experimental, and the study design is characterized as semiquantitative research which belongs to the branch of experimental research. The study sample consisted of paraffin-embedded OLP biopsy samples from the archives of the Department of Oral Medicine/Pathology, School of Dentistry, Aristotle University of Thessaloniki, Greece, during the period 2009-2019. The study sample contained 24 cases of OLP (14 erosive and 10 reticular) and 30 cases of OL (16 cases of moderately and severely dysplastic OL and 14 cases of mildly and non-dysplastic OL). The CSC-related biomarker ALDH1&2 was examined using semiquantitative immunohistochemistry (monoclonal antibody sc-166362, Santa Cruz Biotechnology, Dallas, Texas, USA, 1:100). ALDH1&2 expression was evaluated through a scale of 1 to 3 depending on the percentage of positive epithelial cells and was compared to normal epithelium as well as cases of OL (the most prominent OPMD). The statistical analysis was performed with the Pearson chi-square test and the significance level was set at p≤0.05.

Results: The cytoplasmic staining of ALDH1&2 was observed mostly in the epithelial cells of the basal layer of the epithelium of OLP. Overall, this expression was significantly increased compared to normal epithelium. In addition, statistically significantly higher expression of ALDH1&2 was observed in the erosive form of OLP. Interestingly, this OLP positivity was higher compared to mild and non-dysplastic leukoplakias (p<0.001).

Conclusions: ALDH1&2 is a confirmed CSC marker that was found to be clearly increased in OLP and characteristically in erosive OLP epithelium for the first time. Noteworthy, it was more prominent in erosive OLP rather than in mildly and non-dysplastic OL. Whether this pattern of expression raises the red flag of an early epithelial “CSC” phenotype in OLP or that ALDH1&2 expression indicates a response to the OLP inflammatory process requires further investigation.

## Introduction

The term “oral potentially malignant disorder (OPMD)” is attributed to oral mucosal lesions which exhibit an increased risk for malignant transformation compared to healthy mucosa [1]. This term tends to replace the previous terms of precancerous and premalignant lesions in literature. Oral cancers mostly, but not exclusively, arise from preexisting OPMDs [1]. The most common oral OPMD is oral leukoplakia (OL) [2]. Oral lichen planus (OLP) and oral lichenoid reactions (OLRs) are also considered OPMDs [3]. OLP and OLR share the same clinical characteristics, and their histological features are not easily recognizable. The transition from OPMDs to oral cancer has been hypothesized to be partially mediated by cancer stem cells (CSCs). These cells are implicated in the phenomena of tumor initiation, metastasis, drug resistance, and relapse [4]. The identification of CSCs is achieved through the detection of (combined) specific CSC biomarkers [5]. The presence of CSCs in OL is also well established, but although the identification of CSC biomarkers has proven successful, relevant research remains ongoing [6]. The premalignant nature of OLP and OLR due to dental restorative materials [7] is still under investigation, and the identification of CSC biomarkers could be critically important. OLP/OLR is an immunologically mediated disease, and as such, the lymphocytic infiltration of almost exclusively T-cells plays a critical role in its development. The majority of subepithelial and intraepithelial lymphocytes are located adjacent to damaged basal keratinocytes and colocalized with apoptotic keratinocytes which activated CD8+lymphocytes. The majority of lymphocytes in the lamina propria are CD4+helper T-cells [8-10]. Stemness markers are aldehyde dehydrogenases (ALDH), a group of enzymes that catalyze the oxidation of aldehydes [11]. ALDH is increasingly used as a biomarker of CSCs in squamous cell carcinoma due to the fact that cells positive for it exhibit phenotypic plasticity, generate typical cancer cell spheres and islets in vitro, and exhibit self-renewal. ALDH increases the likelihood of cancer recurrence and is linked to the development of chemo- and radiotherapy resistance [11]. The aim of this study was to investigate the altered expression of a characteristic CSC marker (ALDH1&2) that may indicate the potentially malignant nature of OLP and OLRs. ALDH1&2 was used to compare OLP and OLR cases to the group of OL and to the group of the normal epithelium. The main hypothesis of this study is that OLP/OLR expresses similar CSC biomarkers to leukoplakia and, therefore, belongs to the OPMD.

## Materials and methods

The study type is experimental, and the study design is characterized as semiquantitative research which belongs to the branch of experimental research. Paraffin-embedded samples of 24 cases of the group of OLP, 30 cases of the group of OL, and five cases of the group of normal oral epithelium were used for the immunohistochemical study. The samples were retrieved from the archives of the Department of Oral Medicine/Pathology, School of Dentistry, Aristotle University of Thessaloniki, Greece, during the period 2009-2019. The inclusion criteria were the presence of an adequate quantity of paraffin-embedded tissue in order to successfully perform the immunohistochemical staining, the balanced participation of both male and female patients, and the balanced distribution of the locations of the lesions involved (tongue, cheek, lips, floor of the mouth, gingiva, corner of the mouth). The exclusion criterion was the inadequate quantity of paraffin-embedded tissue. The study was conducted in accordance with the guidelines of the Research and Ethics Committee of Aristotle University, School of Dentistry, and the Helsinki II declaration. The present study was approved by the Ethics Committee of the School of Dentistry, Aristotle University of Thessaloniki, Greece (Nr 8/03.07.2019). The lichen planus group was further divided into the reticular lichen planus group and the erosive lichen planus group as the main clinical forms of OLP/OLR. The leukoplakia group was further divided into the moderately and severely dysplastic leukoplakia group and the mildly dysplastic and non-dysplastic leukoplakia group according to the WHO 2005 binary classification system for OL. The samples included 10 samples of the reticular lichen planus group, 14 samples of the erosive lichen planus group, 16 samples of the moderately and severely dysplastic leukoplakia group, 14 samples of the mildly dysplastic and non-dysplastic leukoplakia group, and five samples of the normal oral epithelium group (Table 1). The group of the normal epithelium functions as the first control group to illustrate the differences between OLP/OLR and the normal epithelium, whereas the group of the leukoplakia functions as the second control group to illustrate the differences and similarities between OLP/OLR and leukoplakia, an OPMD.

**Table 1:** Overview of the samples including the grouping, the coding, the location of the lesions, and the age and gender of the respective patients

Patients	Category	Location	Gender	Age
1	Lichen planus/reticular	Tongue	Male	57
2	Lichen planus/reticular	Tongue	Male	77
3	Lichen planus/reticular	Tongue	Female	21
4	Lichen planus/reticular	Tongue	Female	50
5	Lichen planus/reticular	Tongue	Female	57
6	Lichen planus/reticular	Cheek	Female	72
7	Lichen planus/reticular	Tongue	Female	38
8	Lichen planus/reticular	Cheek	Male	73
9	Lichen planus/reticular	Cheek	Female	49
10	Lichen planus/reticular	Cheek	Female	42
11	Lichen planus/erosive	Interdental papilla	Female	77
12	Lichen planus/erosive	Cheek	Female	77
13	Lichen planus/erosive	Tongue	Female	59
14	Lichen planus/erosive	Cheek	Female	54
15	Lichen planus/erosive	Palate	Female	55
16	Lichen planus/erosive	Cheek	Female	49
17	Lichen planus/erosive	Cheek	Female	72
18	Lichen planus/erosive	Cheek	Female	76
19	Lichen planus/erosive	Cheek	Female	58
20	Lichen planus/erosive	Cheek	Female	64
21	Lichen planus/erosive	Cheek	Male	56
22	Lichen planus/erosive	Cheek	Male	56
23	Lichen planus/erosive	Gingiva	Female	26
24	Lichen planus/erosive	Cheek	Female	37
25	Leukoplakia/moderately and severely dysplastic	Tongue	Female	44
26	Leukoplakia/moderately and severely dysplastic	Tongue	Female	60
27	Leukoplakia/moderately and severely dysplastic	Tongue	Male	58
28	Leukoplakia/moderately and severely dysplastic	Tongue	Female	67
29	Leukoplakia/moderately and severely dysplastic	Tongue	Female	62
30	Leukoplakia/moderately and severely dysplastic	Cheek	Male	66
31	Leukoplakia/moderately and severely dysplastic	Cheek	Male	67
32	Leukoplakia/moderately and severely dysplastic	Tongue	Male	43
33	Leukoplakia/moderately and severely dysplastic	Gingivobuccal sulcus	Female	75
34	Leukoplakia/moderately and severely dysplastic	Tongue	Male	50
35	Leukoplakia/moderately and severely dysplastic	Gingivobuccal sulcus	Male	59
36	Leukoplakia/moderately and severely dysplastic	Tongue	Male	75
37	Leukoplakia/moderately and severely dysplastic	Tongue	Male	64
38	Leukoplakia/moderately and severely dysplastic	Tongue	Male	45
39	Leukoplakia/moderately and severely dysplastic	Palate	Male	72
40	Leukoplakia/moderately and severely dysplastic	Tongue	Female	84
41	Leukoplakia/mildly dysplastic and non-dysplastic	Tongue	Female	61
42	Leukoplakia/mildly dysplastic and non-dysplastic	Lip	Female	38
43	Leukoplakia/mildly dysplastic and non-dysplastic	Tongue	Male	46
44	Leukoplakia/mildly dysplastic and non-dysplastic	Gingiva	Female	12
45	Leukoplakia/mildly dysplastic and non-dysplastic	Tongue	Female	45
46	Leukoplakia/mildly dysplastic and non-dysplastic	Tongue	Male	67
47	Leukoplakia/mildly dysplastic and non-dysplastic	Cheek	Female	60
48	Leukoplakia/mildly dysplastic and non-dysplastic	Tongue	Female	68
49	Leukoplakia/mildly dysplastic and non-dysplastic	Tongue	Male	69
50	Leukoplakia/mildly dysplastic and non-dysplastic	Tongue	Female	68
51	Leukoplakia/mildly dysplastic and non-dysplastic	Cheek	Female	58
52	Leukoplakia/mildly dysplastic and non-dysplastic	Cheek	Female	61
53	Leukoplakia/mildly dysplastic and non-dysplastic	Cheek	Male	75
54	Leukoplakia/mildly dysplastic and non-dysplastic	Corner of the mouth	Male	37
55	Normal	Tongue	Female	49
56	Normal	Cheek	Male	81
57	Normal	Cheek	Female	59
58	Normal	Tongue	Male	69
59	Normal	Tongue	Female	72

For the immunohistochemical method, the CSC protein-biomarker anti-ALDH1&2 (sc-166362, Santa Cruz Biotechnology, Dallas, Texas, USA) and the Dako Envision Flex+ system (Dako Denmark A/S, Glostrup Municipality, Glostrup, Denmark) were used. The evaluation of the immunostaining is quantitative. The quantitative evaluation of the immunostaining corresponds to the percentage of positive cells, which is then classified into a scale ranging from 1 to 3 as follows: (1) the presence of positive cells in one-third of the epithelium, (2) the presence of positive cells in two-thirds of the epithelium, (3) and the presence of positive cells in more than two-thirds of the epithelium. More analytically, the quantitative evaluation of immunostaining is as follows: The evaluation of the cytoplasmic staining of ALDH1&2 is obtained as a histochemical score by calculating the percentage of positive cells and then classifying this percentage into a scale of 1-3 (Table 2). The staining is generally deemed to be successful when the cytoplasm, membrane, or nucleus is colored brown.

**Table 2:** Histochemical score of ALDH1&2

Percentage	Score
0-5%	0
6-35%	1
36-70%	2
>71%	3

Statistical analysis was performed using SPSS Statistics version 25.0 (IBM Corp. Released 2017. IBM SPSS Statistics for Windows, Version 25.0. Armonk, NY: IBM Corp.) with Pearson chi-square test and Fisher’s exact test depending on the sample size. The significance level was set at 0.05 (p=0.05).

## Results

Regarding the staining of ALDH1&2 (Table 3), seven samples of the reticular lichen planus group were scored as 1, and three samples of the reticular lichen planus group were scored as 2. Four samples of the erosive lichen planus group were scored as 1, nine samples of the erosive lichen planus group were scored as 2, and one sample of the erosive lichen planus group was scored as 3. One sample of the moderately and severely dysplastic leukoplakia group was scored as 0, four samples of the moderately and severely dysplastic leukoplakia group were scored as 1, five samples of the moderately and severely dysplastic leukoplakia group were scored as 2, and six samples of the moderately and severely dysplastic leukoplakia group were scored as 3. All the samples of the mildly dysplastic and non-dysplastic leukoplakia group were scored as 1. Three samples of the normal oral epithelium group were scored as 1, and two samples of the normal oral epithelium group were scored as 2.

**Table 3:** Summary of each sample with the respective histochemical scores

PATIENTS	ALDH1&2
1	2
2	1
3	1
4	1
5	2
6	1
7	2
8	1
9	1
10	1
11	2
12	2
13	2
14	2
15	2
16	2
17	2
18	1
19	2
20	1
21	2
22	3
23	1
24	1
25	3
26	2
27	3
28	3
29	3
30	2
31	3
32	3
33	2
34	2
35	2
36	1
37	1
38	1
39	1
40	0
41	1
42	1
43	1
44	1
45	1
46	1
47	1
48	1
49	1
50	1
51	1
52	1
53	1
54	1
55	1
56	1
57	1
58	2
59	2

**Figure 1:**
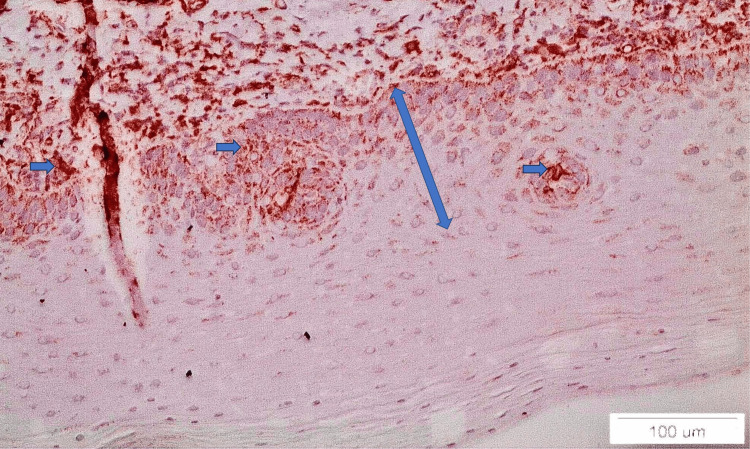
ALDH1&2 staining in a case of reticular lichen planus (x20). The lower third of the epithelium is stained (double-edged arrow) and positively stained cells are noticed (blue arrows)

**Figure 2:**
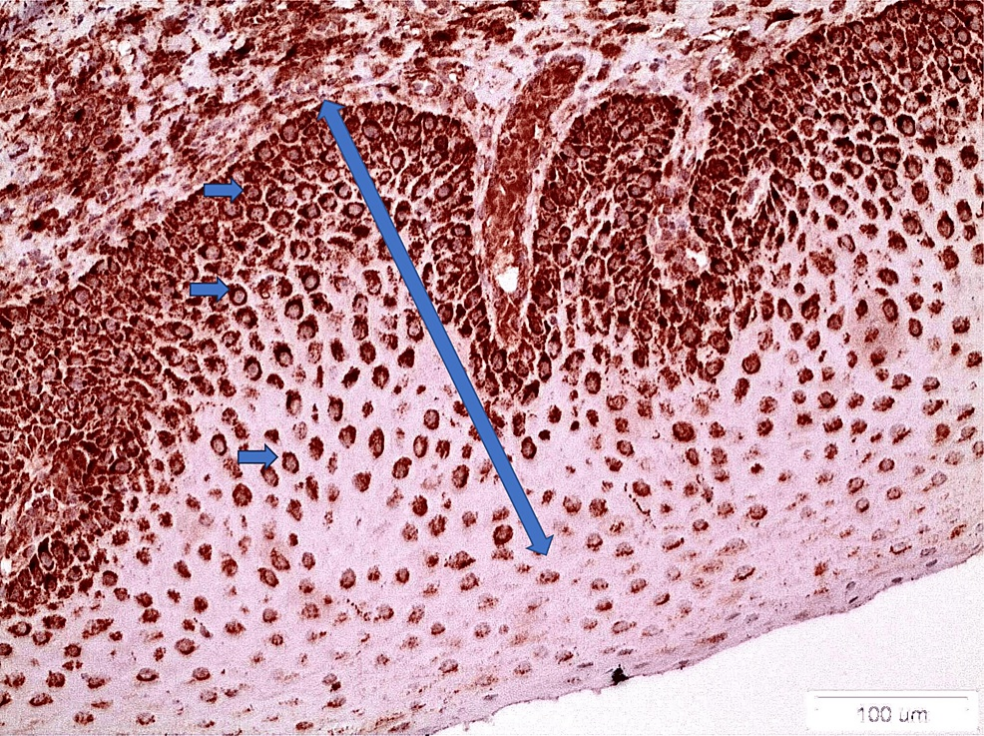
ALDH1&2 staining in a case of erosive lichen planus (x20). The staining of the lower and middle third of the epithelium is noticed (double-edged arrow). In addition, positively stained cells are noticed (blue arrows)

The microscopic images of the reticular lichen planus group and the erosive lichen planus group correspond to Figures 1-2.

The research question was whether the histochemical scores of ALDH1&2 were statistically significantly altered among OLP and OL, their respective subtypes, and normal oral epithelium. Based on the histochemical scores and the statistical analysis, statistically significantly higher expression of ALDH1&2 was noticed in the leukoplakia group than in the lichen planus group (Pearson chi-square test, p=0.035) and in the erosive lichen planus group than in the mildly and non-dysplastic leukoplakia group (Pearson chi-square test, p<0.001).

## Discussion

The presence of CSCs in OPMD induces tumorigenesis [12]. The CSC markers are utilized to identify the CSC populations and are divided into two distinct subgroups: the embryonic stem cell markers and the cancer stem markers [12]. CSCs express cancer stem markers that regulate the molecular programs that underlie the stem cell state. ALDH constitutes a stemness marker, essential for the maintenance of the stem cell state. ALDH is a cytosolic enzyme responsible for catalyzing the pyridine nucleotide-dependent oxidation of aldehydes to carboxylic acids [13]. The aim of this experimental study was to investigate whether lichen planus involves the expression of distinct CSC biomarkers. This goal was addressed by comparing lichen planus samples to the normal oral epithelium (which constitutes the first control group) and leukoplakia (which constitutes the second control group). CSCs mediate the transition of potentially malignant lesions to oral squamous cell carcinoma, and therefore, their presence may support the hypothesis that a disorder is potentially malignant. The evidence so far, regarding the biomarker ALDH1&2, includes the following observations: ALDH has increasingly been used as a CSC marker in oral cavity squamous cell carcinoma (OCSCC), with ALDH+ cells demonstrating plasticity with the ability to form tumor spheres in serum-free media as well as having the ability to generate ALDH cells in vitro [14]. ALDH1 appears to be of particular importance [15]. ALDH1 mediates the malignant transformation of OL to OCSCC since ALDH1+ leukoplakia is more than three times more likely to develop OCSCC [16]. Overexpression of ALDH1 is correlated with nodal metastasis [17]. ALDH+ subpopulation expresses many known CSC-related genes not seen in the ALDH− population [14]. ALDH1+ cells show radioresistance and express the EMT-TF. Snail knockdown downregulates ALDH1 expression, inhibits CSC properties, and results in decreased tumorigenicity [18]. ALDH1A1 is expressed more in OL than in OSCC while ALDH2 is expressed in all OSCC cases [19]. The evidence, originating from our study, indicates that the moderately and severely dysplastic leukoplakias, a well-known potentially malignant oral disorder, expressed the biomarker ALDH1&2 similarly to the erosive lichen planus. A logical assumption to be drawn is that the two entities behave similarly on a molecular basis despite the factor of inflammation in OLP. This conclusion is in accordance with the study of Feng et al. that ALDH1 expression was significantly correlated with the risk of malignant transformation [20], enhancing the hypothesis that erosive lichenoid lesions are similar to moderate and severe dysplastic leukoplakias regarding their prognosis. Mansourian et al. stated that erosive lichenoid lesions have an increased risk of malignant transformation and, in such cases, ALDH1 was overexpressed [21]. On the other hand, the mildly and non-dysplastic leukoplakia group expressed ALDH1&2 similarly to the reticular lichen planus group. A logical assumption to be drawn is that the oral epithelium of the two entities may share partially similar a molecular basis. ALDH1&2 is not statistically significantly different between the erosive lichen planus group and the reticular lichen planus group. This conclusion contradicts the study of Mansourian et al. [21] where the researchers noticed that ALDH1 was much more highly expressed in erosive OLP in comparison to the reticular OLP, thus supporting the prognostic value of ALDH1 in lichenoid lesions. ALDH is also normally expressed in the mitochondria and is upregulated in case of inflammation [22]. OLP and OLR are oral diseases, characterized by the expression of inflammation-related cytokines, triggering an ongoing inflammation [23]. Therefore, it may be the case that the expression of ALDH indicates both the presence of CSCs but also the presence of underlying inflammation. Chronic inflammation constitutes another confounding factor in the appearance of cancer by leading to the expression of other CSC biomarkers [24].

The limitations of our study included the lack of follow-ups of the patients, from whom the tissue specimens were derived, and the lack of study of additional target molecules.

## Conclusions

Moderate and severe dysplastic leukoplakia is a well-known potentially malignant oral disorder and expressed ALDH1&2 similarly to the erosive lichen planus group. The characteristic expression of ALDH1&2 in oral potentially malignant lesions of OLP suggests the presence of CSCs and might imply oral tumorigenesis even in lichenoid lesions. However, the underlying inflammation poses the question of whether the presence of ALDH1&2 indicates exclusively the presence of CSCs or is also influenced by the underlying inflammation. Solid suggestions for future research would be the immunohistochemical staining of more CSC biomarkers to better understand the molecular profile of lichen planus due to dental restorative materials.
